# 10-(2-Eth­oxy-1,3-thia­zol-5-yl)-10-hy­droxy­phenanthren-9(10*H*)-one

**DOI:** 10.1107/S1600536810031004

**Published:** 2010-08-11

**Authors:** Hoong-Kun Fun, Jia Hao Goh, Yang Liu, Yan Zhang

**Affiliations:** a X-ray Crystallography Unit, School of Physics, Universiti Sains Malaysia, 11800 USM, Penang, Malaysia; b School of Chemistry and Chemical Engineering, Nanjing University, Nanjing 210093, People’s Republic of China

## Abstract

In the title compound, C_19_H_15_NO_3_S, the dihydro­phenanthrene unit is not planar, its central ring being distorted towards a sofa conformation. The essentially planar thia­zole ring [maximum deviation = 0.005 (1) Å] is inclined at a dihedral angle of 85.29 (5)° with respect to the mean plane formed through the dihydro­phenanthrene unit. In the crystal structure, pairs of inter­molecular C—H⋯O hydrogen bonds link adjacent mol­ecules into inversion dimers. Inter­molecular O—H⋯N hydrogen bonds further inter­connect these dimers into chains along the *a* axis. The crystal structure is further stabilized by weak inter­molecular C—H⋯π inter­actions involving the thia­zole ring.

## Related literature

For general background to and applications of phenanthrenone derivatives, see: Schuetzle (1983 ▶); Cho *et al.* (2004 ▶); Lim *et al.* (1998 ▶); Sanbongi *et al.* (2003 ▶); Shurygina *et al.* (2008 ▶); Zhang *et al.* (2004 ▶); Lichtenthaler *et al.* (2004 ▶); Cutignano *et al.* (2001 ▶); Williams *et al.* (2001 ▶); DeRoy & Charette (2003 ▶); Yoshimura *et al.* (1995 ▶); Tsuruni *et al.* (1995 ▶); Gao *et al.* (2010 ▶); Shi *et al.* (2010 ▶); Kaleta *et al.* (2006 ▶). For ring conformations, see: Cremer & Pople (1975 ▶). For a closely related phenanthrenone structure, see: Wang *et al.* (2003 ▶). For the stability of the temperature controller used in the data collection, see: Cosier & Glazer (1986 ▶).
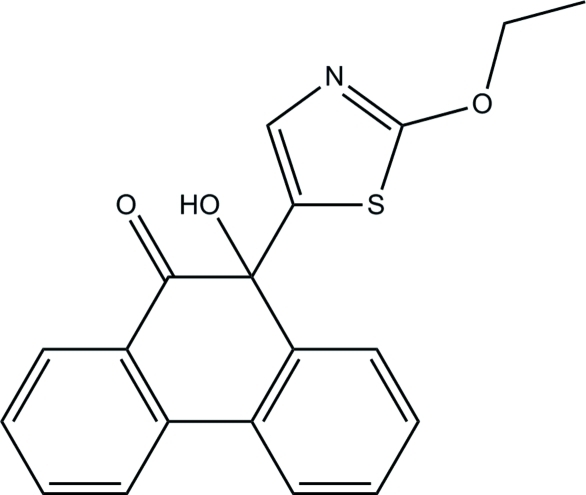


## Experimental

### 

#### Crystal data

C_19_H_15_NO_3_S
*M* *_r_* = 337.38
Triclinic, 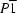

*a* = 7.1386 (4) Å
*b* = 9.6206 (6) Å
*c* = 12.7743 (8) Å
α = 106.863 (2)°
β = 97.746 (2)°
γ = 104.667 (2)°
*V* = 791.41 (8) Å^3^
*Z* = 2
Mo *K*α radiation
μ = 0.22 mm^−1^
*T* = 100 K
0.40 × 0.31 × 0.20 mm

#### Data collection

Bruker APEXII DUO CCD area-detector diffractometer
Absorption correction: multi-scan (*SADABS*; Bruker, 2009 ▶) *T*_min_ = 0.916, *T*_max_ = 0.958
16288 measured reflections
4157 independent reflections
3811 reflections with *I* > 2σ(*I*)
*R*_int_ = 0.023

#### Refinement

*R*[*F* ^2^ > 2σ(*F* ^2^)] = 0.042
*wR*(*F* ^2^) = 0.139
*S* = 1.12
4157 reflections
222 parameters
H atoms treated by a mixture of independent and constrained refinement
Δρ_max_ = 0.96 e Å^−3^
Δρ_min_ = −0.51 e Å^−3^

### 

Data collection: *APEX2* (Bruker, 2009 ▶); cell refinement: *SAINT* (Bruker, 2009 ▶); data reduction: *SAINT*; program(s) used to solve structure: *SHELXTL* (Sheldrick, 2008 ▶); program(s) used to refine structure: *SHELXTL*; molecular graphics: *SHELXTL*; software used to prepare material for publication: *SHELXTL* and *PLATON* (Spek, 2009 ▶).

## Supplementary Material

Crystal structure: contains datablocks global, I. DOI: 10.1107/S1600536810031004/bt5320sup1.cif

Structure factors: contains datablocks I. DOI: 10.1107/S1600536810031004/bt5320Isup2.hkl

Additional supplementary materials:  crystallographic information; 3D view; checkCIF report

## Figures and Tables

**Table 1:** Hydrogen-bond geometry (Å, °) *Cg*1 is the centroid of the thia­zole ring.

*D*—H⋯*A*	*D*—H	H⋯*A*	*D*⋯*A*	*D*—H⋯*A*
O2—H1*O*2⋯N1^i^	0.87 (3)	2.08 (3)	2.8643 (18)	149 (2)
C12—H12*A*⋯O3^ii^	0.93	2.52	3.4395 (18)	170
C4—H4*A*⋯*Cg*1^iii^	0.93	2.70	3.563	155

Symmetry codes: (i) 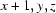; (ii) 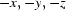; (iii) 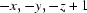.

**Fig. 1.**
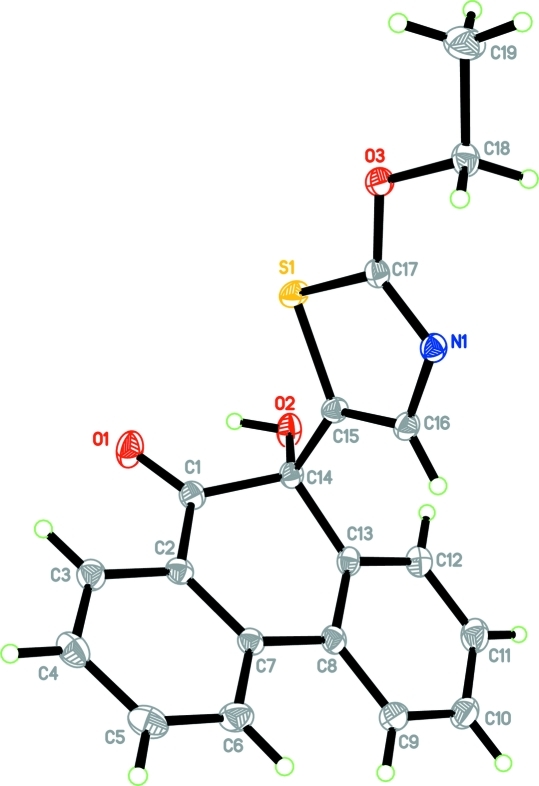
The molecular structure of the title compound, showing the atomic numbering scheme. Displacement ellipsoids are drawn at the 50% probability level. Hydrogen atoms are shown as spheres of arbitrary radius.

**Fig. 2.**
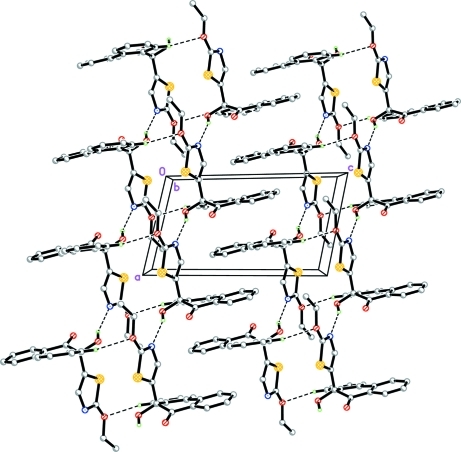
The crystal packing of the title compound, viewed along the b axis, showing the molecules being linked into chains along the a axis.

## supplementary crystallographic information

### Comment

Phenanthraquinone and its derivatives have shown diverse applications and biological activities (Schuetzle, 1983; Cho *et al.*, 2004; Lim *et al.*, 1998; Sanbongi *et al.*, 2003). 9,10-Phenanthraquinone has been used as o-quinone in photoreactions with various species (Shurygina *et al.*, 2008; Zhang *et al.*, 2004; Lichtenthaler *et al.*, 2004). Thiazole-containing compounds, such as the mycothiazole  (Cutignano *et al.*,2001), cystothiazole A (Williams *et al.*,2001; DeRoy & Charette,2003) and WS75624 B (Yoshimura *et al.*,1995; Tsuruni *et al.*,1995) have attracted considerable interest due to their potential application as bio-active species. Synthesis of organic molecules containing thiazole moieties therefore has been of current research interest (Gao *et al.*,2010; Shi *et al.*,2010; Kaleta *et al.*,2006). The title compound which contains phenanthraquinone and thiazole ring may has a potential use in biochemical and pharmaceutical fields. Due to the importance of the phenanthraquinone derivaties, we report in this paper the crystal structure of the title compound.

In the title phenanthraquinone compound (Fig. 1), the 1,2-dihydrobenzene ring (C1/C2/C7/C8/C13/C14) of the 9,10-dihydrophenanthrene ring system (C1-C14) adopts a sofa conformation, with atoms C1 and C14 deviating from the mean plane through the remaning four atoms in opposite directions by 0.1244 (15) and -0.3597 (15) Å, respectively. The puckering parameters are Q = 0.3293 (16) Å, θ = 67.0 (3)° and φ = 317.5 (3)° (Cremer & Pople, 1975). The thiazole ring (C15/C16/N1/C17/S1) is essentially planar, with a maximum deviation of 0.005 (1) Å at atom C17. The mean plane formed through the 9,10-dihydrophenanthrene ring system is approximately perpendicular to the thiazole ring, as indicated by the dihedral angle formed between them being 85.29 (5)°. The geometric parameters are consistent with those observed in closely related 9,10-dihydrophenanthrenone structures (Wang *et al.*, 2003).

In the crystal structure (Fig. 2), adjacent molecules are linked into dimers by pairs of intermolecular C12—H12A···O3 hydrogen bonds (Table 1). These dimers are interconnected by O2—H1O2···N1 hydrogen bonds (Table 1) into two-molecule-wide chains propagating along the *a* axis. Further stabilization of the crystal structure is provided by weak intermolecular C4—H4A···*Cg*1 interactions (Table 1) involving the centroid of the thiazole ring.

### Experimental

The title compound was one of the products from the photoreaction between phenanthraquinone and 2-ethoxylthiazole. The compound was purified by flash column chromatography with ethyl acetate/petroleum ether (1:4) as eluents. X-ray quality single crystals of the title compound were obtained from slow evaporation of an acetone and petroleum ether (1:5) solution. *M.p.* 410–412 K.

### Refinement

The H atom bonded to O was located from difference Fourier map and allowed to refine freely. The remaining hydrogen atoms were placed in their calculated positions, with C—H = 0.93–0.97 Å, and refined using a riding model, with *U*_iso_(H) = 1.2 or 1.5*U*_eq_(C). The rotating group model was applied to the methyl group.

### Crystal data

**Table:**

C_19_H_15_NO_3_S	*Z* = 2
*M**_r_* = 337.38	*F*(000) = 352
Triclinic, *P*1	*D*_x_ = 1.416 Mg m^−^^3^
Hall symbol: -P 1	Mo *K*α radiation, λ = 0.71073 Å
*a* = 7.1386 (4) Å	Cell parameters from 9610 reflections
*b* = 9.6206 (6) Å	θ = 3.0–35.0°
*c* = 12.7743 (8) Å	µ = 0.22 mm^−^^1^
α = 106.863 (2)°	*T* = 100 K
β = 97.746 (2)°	Block, colourless
γ = 104.667 (2)°	0.40 × 0.31 × 0.20 mm
*V* = 791.41 (8) Å^3^	

### Data collection

**Table:**

Bruker APEXII DUO CCD area-detector diffractometer	4157 independent reflections
Radiation source: fine-focus sealed tube	3811 reflections with *I* > 2σ(*I*)
graphite	*R*_int_ = 0.023
φ and ω scans	θ_max_ = 29.0°, θ_min_ = 1.7°
Absorption correction: multi-scan (*SADABS*; Bruker, 2009)	*h* = −9→9
*T*_min_ = 0.916, *T*_max_ = 0.958	*k* = −13→13
16288 measured reflections	*l* = −17→16

### Refinement

**Table:**

Refinement on *F*^2^	Primary atom site location: structure-invariant direct methods
Least-squares matrix: full	Secondary atom site location: difference Fourier map
*R*[*F*^2^ > 2σ(*F*^2^)] = 0.042	Hydrogen site location: inferred from neighbouring sites
*wR*(*F*^2^) = 0.139	H atoms treated by a mixture of independent and constrained refinement
*S* = 1.12	*w* = 1/[σ^2^(*F*_o_^2^) + (0.086*P*)^2^ + 0.3384*P*] where *P* = (*F*_o_^2^ + 2*F*_c_^2^)/3
4157 reflections	(Δ/σ)_max_ < 0.001
222 parameters	Δρ_max_ = 0.96 e Å^−^^3^
0 restraints	Δρ_min_ = −0.51 e Å^−^^3^

### Special details

**Table:**

Experimental. The crystal was placed in the cold stream of an Oxford Cryosystems Cobra open-flow nitrogen cryostat (Cosier & Glazer, 1986) operating at 100.0 (1)K.
Geometry. All esds (except the esd in the dihedral angle between two l.s. planes) are estimated using the full covariance matrix. The cell esds are taken into account individually in the estimation of esds in distances, angles and torsion angles; correlations between esds in cell parameters are only used when they are defined by crystal symmetry. An approximate (isotropic) treatment of cell esds is used for estimating esds involving l.s. planes.
Refinement. Refinement of F^2^ against ALL reflections. The weighted R-factor wR and goodness of fit S are based on F^2^, conventional R-factors R are based on F, with F set to zero for negative F^2^. The threshold expression of F^2^ > 2sigma(F^2^) is used only for calculating R-factors(gt) etc. and is not relevant to the choice of reflections for refinement. R-factors based on F^2^ are statistically about twice as large as those based on F, and R- factors based on ALL data will be even larger.

### Fractional atomic coordinates and isotropic or equivalent isotropic displacement parameters (Å^2^)

**Table:**

	*x*	*y*	*z*	*U*_iso_*/*U*_eq_	
S1	−0.03580 (5)	−0.12894 (4)	0.11117 (3)	0.01623 (12)	
O1	0.39811 (18)	−0.00646 (13)	0.31190 (10)	0.0235 (2)	
O2	0.36598 (16)	0.12494 (13)	0.15415 (9)	0.0185 (2)	
O3	−0.42108 (15)	−0.26001 (11)	0.04040 (8)	0.0161 (2)	
N1	−0.30774 (17)	0.00285 (13)	0.14259 (9)	0.0143 (2)	
C1	0.3195 (2)	0.09376 (16)	0.33170 (11)	0.0154 (3)	
C2	0.2699 (2)	0.15724 (15)	0.44016 (11)	0.0145 (3)	
C3	0.2670 (2)	0.07575 (17)	0.51513 (12)	0.0181 (3)	
H3A	0.2985	−0.0151	0.4963	0.022*	
C4	0.2174 (2)	0.12966 (18)	0.61688 (12)	0.0213 (3)	
H4A	0.2149	0.0754	0.6665	0.026*	
C5	0.1715 (2)	0.26594 (19)	0.64427 (12)	0.0234 (3)	
H5A	0.1340	0.3011	0.7116	0.028*	
C6	0.1809 (2)	0.35008 (17)	0.57240 (12)	0.0204 (3)	
H6A	0.1531	0.4424	0.5932	0.024*	
C7	0.23147 (19)	0.29845 (15)	0.46916 (11)	0.0143 (3)	
C8	0.2552 (2)	0.38983 (16)	0.39351 (11)	0.0153 (3)	
C9	0.2628 (2)	0.54351 (17)	0.42843 (13)	0.0202 (3)	
H9A	0.2493	0.5897	0.5006	0.024*	
C10	0.2901 (2)	0.62833 (17)	0.35718 (14)	0.0228 (3)	
H10A	0.2936	0.7301	0.3816	0.027*	
C11	0.3123 (2)	0.56129 (18)	0.24976 (14)	0.0230 (3)	
H11A	0.3321	0.6184	0.2024	0.028*	
C12	0.3050 (2)	0.40867 (17)	0.21277 (13)	0.0191 (3)	
H12A	0.3189	0.3637	0.1405	0.023*	
C13	0.2769 (2)	0.32302 (16)	0.28379 (12)	0.0151 (3)	
C14	0.2567 (2)	0.15402 (15)	0.23775 (11)	0.0140 (3)	
C15	0.0382 (2)	0.06559 (15)	0.18794 (11)	0.0139 (3)	
C16	−0.1246 (2)	0.11260 (15)	0.19485 (11)	0.0151 (3)	
H16A	−0.1142	0.2136	0.2328	0.018*	
C17	−0.2804 (2)	−0.12792 (15)	0.09691 (11)	0.0134 (2)	
C18	−0.6223 (2)	−0.24987 (16)	0.01743 (12)	0.0168 (3)	
H18A	−0.6257	−0.1734	−0.0177	0.020*	
H18B	−0.6678	−0.2221	0.0866	0.020*	
C19	−0.7522 (2)	−0.40515 (19)	−0.06037 (15)	0.0277 (3)	
H19A	−0.8874	−0.4047	−0.0758	0.042*	
H19B	−0.7438	−0.4801	−0.0256	0.042*	
H19C	−0.7082	−0.4296	−0.1293	0.042*	
H1O2	0.446 (4)	0.075 (3)	0.170 (2)	0.046 (7)*	

### Atomic displacement parameters (Å^2^)

**Table:**

	*U*^11^	*U*^22^	*U*^33^	*U*^12^	*U*^13^	*U*^23^
S1	0.01308 (18)	0.01435 (18)	0.02028 (19)	0.00675 (13)	0.00430 (13)	0.00202 (13)
O1	0.0287 (6)	0.0282 (6)	0.0238 (5)	0.0196 (5)	0.0105 (4)	0.0121 (4)
O2	0.0193 (5)	0.0265 (5)	0.0193 (5)	0.0148 (4)	0.0105 (4)	0.0122 (4)
O3	0.0133 (5)	0.0149 (5)	0.0187 (5)	0.0051 (4)	0.0016 (4)	0.0042 (4)
N1	0.0137 (5)	0.0156 (5)	0.0150 (5)	0.0066 (4)	0.0038 (4)	0.0052 (4)
C1	0.0134 (6)	0.0174 (6)	0.0160 (6)	0.0051 (5)	0.0032 (5)	0.0060 (5)
C2	0.0120 (6)	0.0160 (6)	0.0143 (6)	0.0041 (5)	0.0023 (4)	0.0038 (5)
C3	0.0163 (6)	0.0181 (6)	0.0192 (6)	0.0046 (5)	0.0019 (5)	0.0070 (5)
C4	0.0214 (7)	0.0250 (7)	0.0165 (6)	0.0034 (6)	0.0025 (5)	0.0096 (5)
C5	0.0257 (8)	0.0275 (7)	0.0146 (6)	0.0056 (6)	0.0068 (5)	0.0050 (6)
C6	0.0230 (7)	0.0202 (6)	0.0176 (6)	0.0086 (5)	0.0066 (5)	0.0032 (5)
C7	0.0118 (6)	0.0164 (6)	0.0139 (6)	0.0046 (5)	0.0019 (5)	0.0041 (5)
C8	0.0110 (6)	0.0172 (6)	0.0175 (6)	0.0051 (5)	0.0013 (5)	0.0060 (5)
C9	0.0191 (7)	0.0174 (6)	0.0216 (7)	0.0065 (5)	0.0010 (5)	0.0037 (5)
C10	0.0201 (7)	0.0160 (6)	0.0304 (8)	0.0057 (5)	0.0004 (6)	0.0071 (6)
C11	0.0221 (7)	0.0211 (7)	0.0272 (7)	0.0053 (6)	0.0028 (6)	0.0127 (6)
C12	0.0181 (6)	0.0206 (7)	0.0204 (6)	0.0065 (5)	0.0038 (5)	0.0094 (5)
C13	0.0116 (6)	0.0170 (6)	0.0181 (6)	0.0059 (5)	0.0031 (5)	0.0072 (5)
C14	0.0133 (6)	0.0166 (6)	0.0142 (6)	0.0069 (5)	0.0042 (5)	0.0061 (5)
C15	0.0142 (6)	0.0137 (6)	0.0141 (6)	0.0053 (5)	0.0042 (5)	0.0038 (4)
C16	0.0148 (6)	0.0147 (6)	0.0169 (6)	0.0068 (5)	0.0039 (5)	0.0050 (5)
C17	0.0132 (6)	0.0162 (6)	0.0126 (5)	0.0062 (5)	0.0034 (4)	0.0056 (5)
C18	0.0127 (6)	0.0193 (6)	0.0195 (6)	0.0060 (5)	0.0028 (5)	0.0077 (5)
C19	0.0177 (7)	0.0236 (7)	0.0327 (8)	0.0031 (6)	−0.0008 (6)	0.0020 (6)

### Geometric parameters (Å, °)

**Table:**

S1—C17	1.7335 (14)	C7—C8	1.4821 (19)
S1—C15	1.7430 (14)	C8—C9	1.3994 (19)
O1—C1	1.2161 (18)	C8—C13	1.4100 (19)
O2—C14	1.4105 (16)	C9—C10	1.389 (2)
O2—H1O2	0.87 (3)	C9—H9A	0.9300
O3—C17	1.3308 (16)	C10—C11	1.386 (2)
O3—C18	1.4606 (17)	C10—H10A	0.9300
N1—C17	1.3003 (17)	C11—C12	1.391 (2)
N1—C16	1.3880 (17)	C11—H11A	0.9300
C1—C2	1.4728 (19)	C12—C13	1.3933 (19)
C1—C14	1.5388 (19)	C12—H12A	0.9300
C2—C3	1.4019 (19)	C13—C14	1.5211 (19)
C2—C7	1.4079 (19)	C14—C15	1.5189 (19)
C3—C4	1.382 (2)	C15—C16	1.3543 (19)
C3—H3A	0.9300	C16—H16A	0.9300
C4—C5	1.390 (2)	C18—C19	1.506 (2)
C4—H4A	0.9300	C18—H18A	0.9700
C5—C6	1.387 (2)	C18—H18B	0.9700
C5—H5A	0.9300	C19—H19A	0.9600
C6—C7	1.3984 (19)	C19—H19B	0.9600
C6—H6A	0.9300	C19—H19C	0.9600
			
C17—S1—C15	88.43 (6)	C10—C11—H11A	120.0
C14—O2—H1O2	110.0 (18)	C12—C11—H11A	120.0
C17—O3—C18	115.16 (11)	C11—C12—C13	120.15 (14)
C17—N1—C16	109.10 (11)	C11—C12—H12A	119.9
O1—C1—C2	123.78 (13)	C13—C12—H12A	119.9
O1—C1—C14	119.33 (12)	C12—C13—C8	120.47 (13)
C2—C1—C14	116.81 (12)	C12—C13—C14	118.51 (12)
C3—C2—C7	120.73 (13)	C8—C13—C14	120.90 (12)
C3—C2—C1	118.45 (13)	O2—C14—C15	109.48 (11)
C7—C2—C1	120.80 (12)	O2—C14—C13	111.05 (11)
C4—C3—C2	120.36 (14)	C15—C14—C13	108.38 (11)
C4—C3—H3A	119.8	O2—C14—C1	110.69 (11)
C2—C3—H3A	119.8	C15—C14—C1	105.45 (11)
C3—C4—C5	119.26 (13)	C13—C14—C1	111.60 (11)
C3—C4—H4A	120.4	C16—C15—C14	130.07 (12)
C5—C4—H4A	120.4	C16—C15—S1	109.29 (10)
C6—C5—C4	120.78 (14)	C14—C15—S1	120.62 (10)
C6—C5—H5A	119.6	C15—C16—N1	116.81 (12)
C4—C5—H5A	119.6	C15—C16—H16A	121.6
C5—C6—C7	121.10 (14)	N1—C16—H16A	121.6
C5—C6—H6A	119.5	N1—C17—O3	126.45 (12)
C7—C6—H6A	119.5	N1—C17—S1	116.37 (10)
C6—C7—C2	117.69 (13)	O3—C17—S1	117.18 (10)
C6—C7—C8	122.51 (13)	O3—C18—C19	106.62 (12)
C2—C7—C8	119.73 (12)	O3—C18—H18A	110.4
C9—C8—C13	118.16 (13)	C19—C18—H18A	110.4
C9—C8—C7	122.03 (13)	O3—C18—H18B	110.4
C13—C8—C7	119.79 (12)	C19—C18—H18B	110.4
C10—C9—C8	121.18 (14)	H18A—C18—H18B	108.6
C10—C9—H9A	119.4	C18—C19—H19A	109.5
C8—C9—H9A	119.4	C18—C19—H19B	109.5
C11—C10—C9	120.00 (14)	H19A—C19—H19B	109.5
C11—C10—H10A	120.0	C18—C19—H19C	109.5
C9—C10—H10A	120.0	H19A—C19—H19C	109.5
C10—C11—C12	120.05 (14)	H19B—C19—H19C	109.5
			
O1—C1—C2—C3	−16.1 (2)	C12—C13—C14—O2	30.47 (17)
C14—C1—C2—C3	160.58 (12)	C8—C13—C14—O2	−153.54 (12)
O1—C1—C2—C7	162.22 (14)	C12—C13—C14—C15	−89.82 (15)
C14—C1—C2—C7	−21.08 (18)	C8—C13—C14—C15	86.17 (15)
C7—C2—C3—C4	2.9 (2)	C12—C13—C14—C1	154.49 (12)
C1—C2—C3—C4	−178.75 (13)	C8—C13—C14—C1	−29.51 (17)
C2—C3—C4—C5	−0.3 (2)	O1—C1—C14—O2	−22.47 (18)
C3—C4—C5—C6	−2.0 (2)	C2—C1—C14—O2	160.67 (11)
C4—C5—C6—C7	1.8 (2)	O1—C1—C14—C15	95.84 (15)
C5—C6—C7—C2	0.8 (2)	C2—C1—C14—C15	−81.02 (14)
C5—C6—C7—C8	−176.09 (13)	O1—C1—C14—C13	−146.70 (13)
C3—C2—C7—C6	−3.1 (2)	C2—C1—C14—C13	36.44 (16)
C1—C2—C7—C6	178.58 (12)	O2—C14—C15—C16	−131.81 (15)
C3—C2—C7—C8	173.88 (12)	C13—C14—C15—C16	−10.54 (19)
C1—C2—C7—C8	−4.43 (19)	C1—C14—C15—C16	109.08 (16)
C6—C7—C8—C9	11.4 (2)	O2—C14—C15—S1	50.22 (14)
C2—C7—C8—C9	−165.45 (13)	C13—C14—C15—S1	171.49 (9)
C6—C7—C8—C13	−170.55 (13)	C1—C14—C15—S1	−68.89 (13)
C2—C7—C8—C13	12.60 (19)	C17—S1—C15—C16	−0.46 (10)
C13—C8—C9—C10	0.3 (2)	C17—S1—C15—C14	177.89 (11)
C7—C8—C9—C10	178.36 (13)	C14—C15—C16—N1	−178.05 (12)
C8—C9—C10—C11	−0.6 (2)	S1—C15—C16—N1	0.10 (15)
C9—C10—C11—C12	0.7 (2)	C17—N1—C16—C15	0.48 (17)
C10—C11—C12—C13	−0.5 (2)	C16—N1—C17—O3	179.32 (12)
C11—C12—C13—C8	0.2 (2)	C16—N1—C17—S1	−0.87 (14)
C11—C12—C13—C14	176.17 (13)	C18—O3—C17—N1	7.77 (19)
C9—C8—C13—C12	0.0 (2)	C18—O3—C17—S1	−172.05 (9)
C7—C8—C13—C12	−178.18 (12)	C15—S1—C17—N1	0.80 (11)
C9—C8—C13—C14	−175.96 (13)	C15—S1—C17—O3	−179.37 (11)
C7—C8—C13—C14	5.91 (19)	C17—O3—C18—C19	171.99 (12)

### Hydrogen-bond geometry (Å, °)

**Table:**

Cg1 is the centroid of the thiazole ring.

**Table:**

*D*—H···*A*	*D*—H	H···*A*	*D*···*A*	*D*—H···*A*
O2—H1O2···N1^i^	0.87 (3)	2.08 (3)	2.8643 (18)	149 (2)
C12—H12A···O3^ii^	0.93	2.52	3.4395 (18)	170.
C4—H4A···Cg1^iii^	0.93	2.70	3.563	155

Symmetry codes: (i) *x*+1, *y*, *z*; (ii) −*x*, −*y*, −*z*; (iii) −*x*, −*y*, −*z*+1.
